# Seasonal Differences in the Day-of-the-Week Pattern of Suicide in Queensland, Australia

**DOI:** 10.3390/ijerph10072825

**Published:** 2013-07-08

**Authors:** Chi-kin Law, Diego De Leo

**Affiliations:** Australian Institute for Suicide Research and Prevention, National Centre of Excellence in Suicide Prevention, WHO Collaborating Centre for Research and Training in Suicide Prevention, Mt Gravatt Campus, Griffith University, Queensland 4122, Australia; E-Mail: d.deleo@griffith.edu.au

**Keywords:** day of the week, season, temporal variation, suicide, Australia, Queensland

## Abstract

Various temporal patterns of suicide events, according to time of day, day of week, month and season, have been identified. However, whether different dimensions of time interact has not been investigated. Using suicide data from Queensland, Australia, this study aims to verify if there is an interaction effect between seasonal and day-of-the-week distribution. Computerized suicide data from the Queensland Suicide Register for those aged 15+ years were analyzed according to date of death, age, sex and geographic location for the period 1996–2007. To examine seasonal differences in day-of-the-week pattern of suicide, Poisson regressions were used. A total of 6,555 suicides were recorded over the whole study period. Regardless of the season, male residents of Brisbane had a significantly marked day-of-the-week pattern of suicide, with higher rates between Mondays and Thursdays. When seasonal differences were considered, male residents in Brisbane showed a Monday peak in summer and a wave-shape pattern with a peak on Thursday and a nadir on Saturdays in winter. Whilst males have distinctive peaks in terms of days of the week for summer and winter, females do not show similar patterns.

## 1. Introduction

Temporal variations in suicide rates have attracted attention in research from both clinical and epidemiological perspectives [1]. In an effort to understand and explain the phenomenon, many studies on this topic have mainly focused on the monthly or seasonal patterns, or patterns of the day of the week and time of the day [2]. Literature on day-of-the-week pattern of suicide, mainly from the Northern Hemisphere, has consistently found a peak on Mondays and a nadir on weekends [3,4,5,6]. Other temporal patterns by season, by month, and so forth have also been examined, which identified a particular peak in the number of suicide in late spring or early summer for men, a bimodal distribution pattern with peaks in spring and autumn for women and a nadir in winter months for both sexes [2,5,7,8,9,10,11,12,13,14,15,16,17,18]. However, the possible existence of a relationship between time dimensions (*i.e.*, seasonal and day-of-the-week patterns) has not been previously investigated.

It is of interest to understand more about the possible interaction of these two time dimensions. For example, the day-of-the-week pattern may become more or less pronounced during the seasons previously found to have elevated and reduced risk of suicide. Demonstrating such effects would extend our scientific knowledge of the temporal patterns of suicidal behaviour and may provide useful information for planning more effective population-based suicide prevention strategies [2].

This study aims to fill this gap in knowledge by analyzing the day-of-the-week pattern of suicide by sex and residence across the four seasons of the year using suicide data from Queensland, Australia for the period from 1996 to 2007. As the second largest state of Australia with a population of 4.47 million in 2011, Queensland has the highest incidence of youth suicide in the nation [19] and is a potentially interesting region of the study of temporal variation of suicide [10]. Because previous work by Kalediene and Petrauskiene [4] has shown the day-of-the-week patterns differ significantly between urban and rural residents, suicide mortality of Greater Brisbane (as a proxy of urban population) and the rest of Queensland population (as a proxy for rural population) were separately assessed in the present study.

## 2. Methods

### 2.1. Suicide Mortality Data Source

Suicide data for the period 1996–2007 were retrieved from the Queensland Suicide Register (QSR), which includes all suicide data for the state of Queensland, Australia [20]. Suicides were identified as those with ICD-10 code ranging from X60 to X84 [21]. Deaths by undetermined causes were not included. For the purposes of regional analysis, we divided the state of Queensland into two regions: Greater Brisbane (Statistical Area Level 4: 301–305) and the rest of Queensland (Statistical Area Level 4: 306–319), as specified by the 2011 Australian Statistical Geography Standard [22]. To safeguard privacy, all relevant files were stored in the investigator’s computer and secured with a password lock to restrict any unauthorized access. Ethics approval of the project was granted by the Griffith University Human Research Ethics Committee (GU Ref No: CSR/02/10/HREC) and the Justice Human Research Ethics Committee (CF/12/14310).

### 2.2. Statistical Analysis

Statistical analyses were performed using SPSS for Windows version 20.0. For analyses of seasonal and day-of-the-week patterns of suicide, data were divided into the 4 seasons of the year and the 7 days of the week. Here, we followed the traditional calendar specification in the Southern Hemisphere, which groups December to February as summer, March to May as autumn, June to August as winter and September to November as spring [10,12]. To examine the day-of-the-week variation in suicides and its statistical significance in each season, four separate multinomial Poisson regression models were compiled by fitting the number of suicides for each day of the week with the corresponding time variable [14,23]. To adjust for the differences in suicide rates among different subgroups of the population, we included age, sex, residence (Brisbane and the rest of Queensland), year of incidence and the offset term of population size in the regression equation as the control variables. Population size of Queensland by age, sex and region over the study period was made available from the Australian Bureau of Statistics. The regression Equation (1) is written as follows:

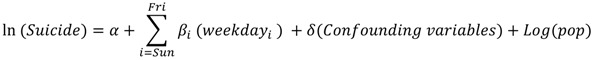
(1)

The season-specific day-of-the-week variation for residents in Brisbane and the rest of Queensland was modeled by comparing the actual frequencies of suicides with the expected values that assume all suicides to be equally distributed. The exponential of the regression coefficient (β) represents the incidence risk ratio (IRR), which describes the multiplicative effect of the corresponding independent variable on the risk [5,24]. A significant IRR indicates a significant difference in the adjusted suicide risk between the corresponding day of the week and the time reference period. In identifying the “high risk time” of suicide, the day of the week with the lowest risk of suicide was used as the reference [4,5,7].

In the next step, subgroups of residents with pronounced day-of-the-week patterns in any season of the year were selected and examined further to see whether there were any significant seasonal differences in terms of their day-of-the-week pattern of suicide. Those subgroups with no pronounced season-specific day-of-the-week variation were excluded. In assessing seasonal difference and its statistical significance on the day-of-the-week pattern of suicide, we grouped the suicide data of the four seasons and re-iterated the Poisson regression by fitting the number of suicides for each sub-group with Equation (2), as specified below:
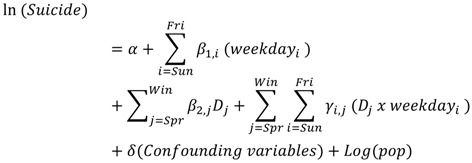
(2)

Here, *D_j_* is a binary variable to indicate seasons of a year. Any season(s) with a significant day-of-the-week pattern of suicide obtained from Equation (1) is assigned a value of 1 while a value of 0 is given for the others. A product term of *D_j_* and weekday was included in the regression model. A significant *γ_i,j_* and *β_j,2_* would indicate that the day-of-the-week distribution at that particular season (*D_j_*) was significantly different from other seasons. For all statistical analyses, a *p*-value smaller than 5% was considered to be statistically significant.

## 3. Results

For the 12-year period, a total of 6,555 suicides aged 15 years and above were identified, 5,188 (79.2%) of which were men and the rest women (n = 1,367 or 20.8%). When analyzed by residence, about 42% (n = 2,767) of suicide cases in Queensland lived in the Greater Brisbane region (Brisbane), with the rest (58%) elsewhere in Queensland (n = 3,788). In line with Cantor* et al.* [10], the number of suicides in Queensland was noted to fluctuate by season, peaking in summer (n = 1,728, 26.4%) and dipping in autumn (n = 1,575, 24.0%) and winter (n = 1,576, 24.0%).

### 3.1. Day-of-the-Week Patterns of Suicides

Poisson regressions revealed that the day-of-the-week pattern of suicide varied considerably across different subgroups of the population (see Table 1). Regardless of season, only male residents of Brisbane had a significantly marked day-of-the-week pattern of suicide, with higher rates on Mondays (IRR = 1.33, *p* ≤ 0.001), Tuesdays (IRR = 1.26, *p* = 0.006), Wednesdays (IRR = 1.19, *p* = 0.044) and Thursdays (IRR = 1.29, *p* = 0.002), when compared with the reference rate for Saturdays. No significant day-of-the-week pattern was found in other sub-groups.

### 3.2. Seasonal Differences in the Day-of-the-Week Pattern of Suicide

Table 1 also depicts the season-specific day-of-the-week patterns of suicide through separate regressions. Male residents in Brisbane and female residents in the rest of Queensland had a marked day-of-the-week pattern of suicide in different seasons. For male residents of Brisbane, the day-of-the-week pattern was significant in summer and winter, with a single peak on Mondays (IRR = 1.62, *p* = 0.002) in summer and higher suicide risk from Sundays to Tuesdays and also on Thursdays in winter. For female residents in the rest of Queensland, a significantly higher rate of suicide on Mondays (IRR = 1.70, *p* = 0.028) was found in spring, whilst the day-of-the-week pattern was not significant in other seasons.

To examine whether the seasonal difference in the day-of-the-week pattern of suicide was statistically significant, Table 2 shows the results of regression Equation (2), which takes both time dimensions (*i.e.*, seasons and days of the week) into consideration. Only male residents in Brisbane had significant seasonal differences in terms of the day-of-the-week pattern of suicide, with a marked single peak on Mondays in summer (*γ_2,sum_* = 0.39, *p* = 0.05) and a wave-shape pattern in winter (with a significantly higher suicide rate on Thursdays (*γ_5,win_* = 0.58, *p* = 0.01) and a lower rate on Saturdays (*β_2,win_* = −0.38, *p* = 0.02). The seasonal difference in the Monday peak was found to be close to significance for female residents in the rest of Queensland (*γ_2,spr_* = 0.54, *p* = 0.06).

**Table 1:** Number and Incidence risk ratios (IRR **^#^**) of suicide by day of the week, season of the year, sex and residence of deceased, Queensland, 1996–2007.

Sex	Residence	Season		Day of the week							Overall
				Sun	Mon	Tue	Wed	Thu	Fri	Sat	
*Male*	*Greater*	*Spring*	*No.*	*75*	*78*	*79*	*92*	*78*	*79*	*71*	*552*
	*Brisbane*		IRR	1.06	1.10	1.11	1.30	1.08	1.11	-	-
		*Summer*	No.	71	107	86	75	84	88	67	578
			IRR	1.08	1.62 *	1.29	1.14	1.27	1.33	-	-
		*Autumn*	No.	72	80	85	71	78	65	72	523
			IRR	1.00	1.10	1.18	0.99	1.08	0.90	-	-
		*Winter*	No.	75	80	75	68	95	70	49	512
			IRR	1.53 *	1.63 *	1.53 *	1.39	1.94 *	1.43	-	-
		*Overall*	No.	293	345	325	306	335	302	259	2165
			IRR	1.14	1.33 *	1.26 *	1.19 *	1.29 *	1.17	-	-
	*Rest of*	*Spring*	*No.*	*112*	*121*	*118*	*102*	*93*	*115*	*106*	*767*
	*Queensland*		IRR	1.06	1.14	1.11	0.95	0.88	1.08	-	-
		Summer	No.	110	122	117	106	105	116	130	806
			IRR	0.85	0.94	0.90	0.82	0.81	0.89	-	-
		Autumn	No.	90	106	105	107	101	118	96	723
			IRR	0.94	1.10	1.09	1.11	1.05	1.23	-	-
		*Winter*	No.	101	111	109	93	104	105	104	727
			IRR	0.97	1.07	1.05	0.89	1.00	1.00	-	-
		*Overall*	No.	413	460	449	408	403	454	436	3023
			IRR	0.95	1.06	1.03	0.93	0.92	1.04	-	-
*Female*	*Greater*	*Spring*	*No.*	*23*	*27*	*13*	*23*	*22*	*23*	*16*	*147*
	*Brisbane*		IRR	1.44	1.69	0.81	1.44	1.37	1.44	-	-
		*Summer*	No.	21	26	25	27	17	20	18	154
			IRR	1.17	1.44	1.39	1.50	0.94	1.11	-	-
		*Autumn*	No.	18	23	20	18	26	17	16	138
			IRR	1.13	1.44	1.25	1.13	1.63	1.06	-	-
		*Winter*	No.	22	19	27	28	26	19	22	163
			IRR	1.00	0.86	1.23	1.27	1.18	0.86	-	-
		*Overall*	No.	84	95	85	96	91	79	72	602
			IRR	1.17	1.32	1.18	1.33	1.26	1.10	-	-
	*Rest of*	*Spring*	*No.*	*22*	*46*	*34*	*29*	*29*	*23*	*27*	*210*
	*Queensland*		IRR	0.81	1.70 *	1.26	1.07	1.07	0.85	-	-
		*Summer*	No.	28	28	27	25	27	23	32	190
			IRR	0.88	0.88	0.84	0.78	0.84	0.72	-	-
		*Autumn*	No.	27	29	25	27	26	28	29	191
			IRR	0.93	1.00	0.86	0.93	0.9	0.93	-	-
		*Winter*	No.	19	35	30	23	19	16	32	174
			IRR	0.59	1.09	0.94	0.72	0.59	0.50	-	-
		*Overall*	No.	96	138	116	104	101	90	120	765
			IRR	0.80	1.15	0.97	0.87	0.84	0.74	-	-

* significant at 5% level; **^#^** Saturday as reference group.

**Table 2:** Regression analysis to examine seasonal differences in the day-of-the-week pattern of suicide for selected groups in Queensland, 1996–2007.

Type of sub-population	Season		Day of the week						
			Sun (*γ_1,j_*)	Mon (*γ_2,j_*)	Tue (*γ_3,j_*)	Wed (*γ_4,j_*)	Thu (*γ_5,j_*)	Fri (*γ_6,j_*)	Sat (*β_2,j_*)
Male residents *	*Summer*	Parameter	0.05	0.39 *	0.12	0.00	0.16	0.28	−0.08
*in Greater*		*p*-value	0.83	0.05	0.56	0.99	0.42	0.16	0.59
*Brisbane*		95% LL	0.70	1.01	0.76	0.67	0.79	0.89	0.69
		95% UL	1.57	2.16	1.66	1.49	1.74	1.96	1.24
	*Winter*	Parameter	0.40	0.40	0.29	0.20	0.58 *	0.35	−0.38 *
		*p*-value	0.07	0.07	0.18	0.37	0.01	0.11	0.02
		95% LL	0.97	0.98	0.87	0.79	1.18	0.92	0.50
		95% UL	2.28	2.27	2.04	1.87	2.70	2.19	0.95
*Female residents*	*Spring*	Parameter	0.02	0.54	0.36	0.29	0.33	0.18	−0.14
*in rest of*		*p*-value	0.94	0.06	0.23	0.35	0.29	0.58	0.53
Queensland **		95% LL	0.54	0.99	0.80	0.73	0.76	0.63	0.57
		95% UL	1.94	3.00	2.57	2.44	2.55	2.28	1.34

* Spring and autumn together as the reference group; ** Summer, autumn and winter together as the reference group.

## 4. Discussion

The results of the present study firstly revealed the risk of suicide varies markedly across the days of the week among male residents in Greater Brisbane, with an excess risk of suicide on weekdays and a peak on Mondays. For females, it is worth noting that a similar day-of-the-week pattern, with higher rates on weekdays in Greater Brisbane and a Monday peak in rest of Queensland, was observed. However, possibly due to the relatively smaller size of female suicides (n = 602 in Greater Brisbane and n = 765 in rest of Queensland) over the study period, our statistical analyses did not confirm these day-of-the-week fluctuations at the 5% significance level. In this regard, a possible control variable is employment status, which is likely to differ between sexes. Unfortunately, data on employment status were not available for a large proportion of suicide cases, and the influence of employment on temporal patterns would need future attention.

Additionally, when looking two-dimensionally at both the day-of-the-week and season of the year in the present analysis, marked differences between summer and winter distribution regarding week of the day were found among male residents in Greater Brisbane. In particular, it is noteworthy that the elevated risk of suicide in summer was mainly contributed by the increase in the number of suicides on Mondays from about 80 in other seasons to 107 (above 30%), which also accounted for the marked Monday peak in the analysis. Similarly, the reduced suicide risk in wintertime was mostly contributed by the decrease in suicides on Saturdays from about 70 in other seasons to only 49 in winter (−30%) over the whole period of study. The considerably smaller number of suicides on Saturdays (the reference day) also accounted for the marked day-of-the-week fluctuation in winter for male residents in Brisbane. Without doubt, this observation gives some new insights on this area, as prior research has never explained why the seasonal fluctuations of suicide risk was highly influenced by the corresponding day-of-the-week variations. This will be a promising avenue for future research. In agreement with the work of Kalediene and Petrauskiene [4], also in the Southern Hemisphere, weekends appear characterized by lower rates of suicide, a fact that seems to underscore the psychosocial importance of non-working days in relation to suicide events.

### Limitations

Findings of this study should be interpreted in light of their limitations. Firstly, one major limitation of this study is the relatively small sample of 6,555 suicide cases, which limits the statistical power of the study (particularly for female subjects). Secondly, date of death, instead of date of the actual suicide act, was used, as this piece of information is not necessarily available for every suicide case in the Register. Delays between suicide and its discovery may inevitably distort the genuine temporal pattern of suicide [25]. Yet, the existing literature suggests that it remains the most acceptable and consistent measure to approximate the occurrence of suicide incidence in epidemiological research [25,26,27]. Further investigations that include analysis of factors such as seasonally-related weather effects [9], length of sunlight [28], suicide method used [14,29,30], substance use, presence of depression [15] and related mood disorders [14] would be required for a more precise understanding of the interaction of different temporal dimensions. In addition, whether the findings of the present study can be generalized to other countries remains to be demonstrated.

## 5. Conclusions

To our knowledge, this is the first study assessing seasonal differences in the day-of-the-week pattern of suicide through Poisson regression. With this analytical approach, the present study provides a more comprehensive picture of the relationship between temporal variation and suicide mortality and also shows an interaction effect of different temporal dimensions. However, whether this interaction effect would be influenced by other socioeconomic factors remains unclear, and consequently would require further attention in future studies. 

Given the larger size of the problem in males, interpretation of data is seemingly more convincing in these subjects. Overall, Mondays confirm their capacity to concentrate more suicide events, particularly in summer. Apparently, the reduced suicide risk in wintertime is mostly contributed by the decrease in suicide deaths on Saturdays. 
